# The Restriction–Modification Systems of *Clostridium carboxidivorans* P7

**DOI:** 10.3390/microorganisms11122962

**Published:** 2023-12-12

**Authors:** Patrick Kottenhahn, Gabriele Philipps, Boyke Bunk, Cathrin Spröer, Stefan Jennewein

**Affiliations:** 1 Department of Industrial Biotechnology, Fraunhofer Institute for Molecular Biology and Applied Ecology IME, 52074 Aachen, Germany; 2 Department of Biology, RWTH Aachen University, 52074 Aachen, Germany; 3 Department Bioinformatics and Databases, Leibniz Institute DSMZ-German Culture Collection for Microorganisms and Cell Cultures, 38124 Braunschweig, Germany

**Keywords:** DNA methylation, restriction enzyme, restriction–modification system, *Clostridium*, genetic transformation

## Abstract

*Clostridium carboxidivorans* P7 (DSM 15243) is a bacterium that converts syngas (a mixture of CO, H_2_, and CO_2_) into hexanol. An optimized and scaled-up industrial process could therefore provide a renewable source of fuels and chemicals while consuming industry waste gases. However, the genetic engineering of this bacterium is hindered by its multiple restriction–modification (RM) systems: the genome of *C. carboxidivorans* encodes at least ten restriction enzymes and eight methyltransferases (MTases). To gain insight into the complex RM systems of *C. carboxidivorans*, we analyzed genomic methylation patterns using single-molecule real-time (SMRT) sequencing and bisulfite sequencing. We identified six methylated sequence motifs. To match the methylation sites to the predicted MTases of *C. carboxidivorans*, we expressed them individually in *Escherichia coli* for functional characterization. Recognition motifs were identified for all three Type I MTases (C**A**YNNNNNCTGC/GC**A**GNNNNNRTG, CC**A**NNNNNNNNTCG/CG**A**NNNNNNNNTGG and GC**A**NNNNNNNTNNCG/CGNN**A**NNNNNNNTGC), two Type II MTases (GATA**A**T and CRAAA**A**R), and a single Type III MTase (GAA**A**T). However, no methylated recognition motif was found for one of the three Type II enzymes. One recognition motif that was methylated in *C. carboxidivorans* but not in *E. coli* (AGA**A**GC) was matched to the remaining Type III MTase through a process of elimination. Understanding these enzymes and the corresponding recognition sites will facilitate the development of genetic tools for *C. carboxidivorans* that can accelerate the industrial exploitation of this strain.

## 1. Introduction

Synthesis gas (syngas) is a promising feedstock for the production of platform chemicals from sources such as municipal waste, industrial process gases, or gasified biomass [1,2,3,4,5]. This could reduce our dependence on fossil resources while limiting greenhouse gas emissions by capturing and using the carbon contained in these gases. So-called acetogenic bacteria can use syngas as their sole carbon and energy source and can convert it into various organic acids and alcohols. However, *Clostridium carboxidivorans* P7 can synthesize medium-chain alcohols such as butanol and hexanol from syngas, which can be used as fuels or as raw materials for the synthesis of plastics and chemicals. Recent developments combining medium and process optimization have already resulted in yields in the range of several grams of hexanol/L [6,7,8].

One barrier hindering the optimization of medium-chain alcohol production is the genetic inaccessibility of *C. carboxidivorans*. Although one study reported four transgenic *C. carboxidivorans* strains with improved ethanol and butanol yields, gene transfer was achieved through conjugation rather than standard electro-transformation procedures because the latter appeared to be inhibited by endogenous restriction–modification (RM) systems [9]. RM systems are ubiquitous in bacteria and are often considered as barriers to genetic engineering and transformation, with a prominent example being bacteria from the genus *Clostridium* [10,11,12,13,14]. They act as an innate immune system for bacteria, restricting phage infection of the host cell [15,16]. Typical RM systems consist of restriction enzymes (restriction endonucleases) that recognize and/or cleave DNA at a particular site and methyltransferases (MTases) that protect the DNA from cleavage by modifying the bases. Type I systems involve ATP-dependent enzyme complexes consisting of two MTase units (M), two restriction endonuclease units (R), and one specificity peptide (S), which selectively target asymmetric, non-palindromic sites consisting of six to seven specific bases interrupted by five to eight variable bases [17]. The actual cleavage occurs up to several kb away from the recognition site, and cleavage results in the complete degradation of DNA containing an unmethylated recognition sequence [18,19]. Type II systems are the most widely used in molecular biology because the R and M components are separate enzymes that target the same site, which is specific and palindromic [20]. The typical modifications are m^6^A, m^5^C, or m^4^C [17]. In Type III systems, the R and M components form a tetrameric complex that recognizes an asymmetric target site and cleavage occurs downstream from the unmethylated site, although digestion is usually incomplete [21,22]. In contrast to Types I, II, and III, Type IV systems consist of restriction enzymes without a corresponding MTase and only cut methylated DNA motifs. Some Type IV enzymes have specific and precise cleavage sites whereas others are non-specific and variable [17]. In summary, Type I–III systems restrict DNA lacking the host’s native methylation pattern, whereas Type IV systems restrict foreign methylation patterns [23].

The genus *Clostridium* (and even individual species within it) features diverse strains ranging from those without RM systems, which are easy to manipulate, to strains with many active RM systems spanning several different types, which are genetically inaccessible [12,14,24,25]. Inconvenient RM systems can be circumvented by ensuring the absence of recognition sites in plasmids used for transformation [26] or by using donor strains with compatible methylation patterns [14,27]. However, neither method is suitable if the methylation patterns of the recipient strain are unknown. Genome sequencing can be used to predict the presence of MTase genes, allowing the creation of a donor strain expressing the recipient’s native methyltransferases, but this approach becomes increasingly complex if the host possesses multiple RM systems.

Herein, we characterized the RM systems of *C. carboxidivorans* by screening the genome for putative RM genes and analyzing the methylation status of *C. carboxidivorans* genomic DNA using PacBio and bisulfite sequencing. The genes encoding each MTase (and specificity unit, where applicable) were cloned and expressed in *Escherichia coli* to determine the induced methylation patterns. This enabled us to match each enzyme to the corresponding recognition motif.

## 2. Materials and Methods

### 2.1. Strains and Cultivation Conditions

*Clostridium carboxidivorans* P7 (DSM 15243) was obtained from the DSMZ (Braunschweig, Germany). The cells were grown heterotrophically in modified minimal medium ATCC 1754 [7] with an oxygen-free atmosphere (5% H_2_, 10% CO_2_, and 85% N_2_) at 30 °C without agitation in a Whitley A55 Anaerobic Workstation (Don Whitley Scientific, Herzlake, Germany). *Escherichia coli* NEB 5-alpha cells (C2987H; New England Biolabs, Ipswich, MA, USA) were used for plasmid propagation and were incubated at 30 °C in a rotary shaker at 150 rpm. For the cultivation of pMT_Solo strains based on pCDFDuet-1, we added spectinomycin (100 mg mL^−1^ dissolved in water) to the LB medium, with a final concentration of 100 µg mL^−1^. The *E. coli* strains used in this study are listed in Table A1.

### 2.2. Plasmid Construction, Sequencing and Transformation

MTase genes and specificity subunit genes (where present) were amplified from *C. carboxidivorans* genomic DNA using Q5 High-Fidelity DNA Polymerase (New England Biolabs) and the specific primers listed in Table A2. The amplicons were inserted into vector pCDFDuet-1 under the control of the inducible T7 promoter. For the analysis of single MTases, Gibson assembly sites were selected so that only one expression site remained in the pCDFDuet-1 vector, enabling the construction of pMT-Solo plasmids. Plasmids assembled through Gibson assembly were introduced into chemically competent *E. coli* NEB 5-alpha cells, which were spread on LB agar plates (Carl Roth, Karlsruhe, Germany) with the appropriate antibiotics and incubated at 30 °C. Single colonies were then transferred to liquid LB medium (Carl Roth) supplemented with 100 µg mL^−1^ spectinomycin, and the cultures were used for plasmid isolation and the preparation of glycerol stocks for storage at −80 °C. Plasmids were isolated using the NucleoSpin Plasmid Mini kit (Macherey-Nagel, Düren, Germany). Plasmid integrity was verified through restriction digestion and in-house Sanger sequencing and/or sequencing using Microsynth Seqlab (Göttingen, Germany). Plasmid sequencing reads were assembled and confirmed using the CLC workbench.

### 2.3. Methylation Analysis

Methylation sites were identified using PacBio SMRT sequencing. *C. carboxidivorans* cells were grown as described above and harvested in the late exponential to early stationary growth phase. *E. coli* NEB 5-alpha cells were grown overnight in LB medium at 30 °C while being shaken at 150 rpm. The medium contained 100 µg mL^−1^ spectinomycin for the selection of pCDFDuet-1 plasmids and 2 mM isopropyl-β-D-thiogalactopyranoside (IPTG) for induction. *E. coli* NEB 5-alpha does not encode a T7 polymerase gene in its genome. However, the T7 promoter region is expected to be accessible to native polymerases. Additionally, pCDFDuet-1 contains cryptic -35 and cryptic -10 promoter boxes adjacent to the T7 promoter, resulting in low-level expression of the MTases. Cells were harvested in the mid-to-late exponential growth phase. Genomic DNA was isolated from *E. coli* using the standard protocol of the NucleoSpin gDNA Mini kit (Macherey-Nagel). For *C. carboxidivorans*, we used the isolation protocol for hard-to-lyse bacteria according to the manufacturer’s recommendations. Genomic DNA was stored at 4 °C and sent to the DSMZ in a cooled overnight parcel for PacBio single molecule real-time (SMRT) sequencing, which can detect the presence of m^6^A and m^4^C [28,29]. 

SMRTbell template libraries were prepared by following the Procedure & Checklist—Preparing Multiplexed Microbial Libraries Using SMRTbell Express Template Prep Kit 2.0 (Pacific Biosciences, Menlo Park, CA, USA). Briefly, 10 kb libraries were prepared by shearing 1 µg of genomic DNA in g-tubes (Diagenode, Denville, NJ, USA) according to the manufacturer’s instructions. The DNA was end-repaired and ligated to barcoded adapters using the SMRTbell Express Template Prep Kit 2.0. Samples were pooled as recommended by the Microbial Multiplexing Calculator. Conditions for primer annealing and the binding of polymerase to the purified SMRTbell template were assessed using the SMRT-Link calculator. Three genomic libraries were sequenced on a Sequel IIe device (Pacific Biosciences) taking one 15 h movie per SMRT cell. One SMRT cell was used for *C. carboxidivorans* and one was used for *E. coli*.

For bioinformatic analysis, all datasets were processed using the Base Modification Analysis Protocol in SMRT-Link 10.0.0.108728. Essentially, the detection of base modifications is based on a (statistical) increase in the inter-pulse duration (IPD) values. The details are described by Feng et al. 2013 [30]. The genomes of *C. carboxidivorans* P7 (RefSeq NZ_CP011803.1) and *E. coli* K-12 NEB 5-alpha (GenBank CP017100.1) were used as references. We applied a modification threshold (Qmod) score of 50 if not stated otherwise. 

To verify the absence of m^5^C methylation in *C. carboxidivorans*, genomic DNA was sent to CD Genomics (CD Biosciences, New York, NY, USA) for bisulfite conversion using the Bisulfite-Seq Library Prep Kit (Acegen, Shenzhen, China) followed by sequencing on an Illumina NovaSeq device in PE150 mode. Briefly, 1 μg of genomic DNA was fragmented through sonication (200–400 bp mean size), followed by end-repair, 5′ phosphorylation, 3′-dA-tailing, and ligation to methylated adapters. The methylated adapter-ligated DNAs were purified using 0.8× Agencourt AMPure XP magnetic beads before bisulfite conversion using the ZYMO EZ DNA Methylation-Gold Kit (Zymo Reasearch, Irvine, CA, USA). The converted DNA was amplified with 25 μL KAPA HiFi HotStart Uracil+ ReadyMix (2X) and 8-bp index primers (final concentration 1 μM each). The library quality was confirmed using an Agilent 2100 Bioanalyzer and quantified using a Qubit fluorometer with the Quant-iT dsDNA HS Assay Kit (Invitrogen, Thermo Fisher Scientific, Waltham, MA, USA). The library was then sequenced using an Illumina HiSeq X ten platform in PE150 mode. 

### 2.4. RNA Isolation and Semi Quantitative RT-PCR

Cells were grown in 50 mL of modified ATCC 1754 medium with fructose in a 500 mL bottle to OD = 0.8 at 30 °C in a Don Whitley anaerobic workstation. The cells were harvested through centrifugation at 4 °C and the pellet was stored at −80 °C. RNA was isolated using the Macherey–Nagel RNA Isolation Kit (with DNase digest) and was reverse transcribed to cDNA before semi-quantitative PCR using the Qiagen QuantiTect Reverse Transcription Kit (with a second DNase digest prior to reverse transcription), Phusion^®^ High-Fidelity DNA Polymerase, and primers specific to each RM component gene (Table A3). The annealing temperature was set to 55 °C, with elongation for 30 s over 35 amplification cycles. Genomic DNA was used as the positive control, total RNA after DNA digestion was used as the negative control, and cDNA was used as the test sample. 

## 3. Results and Discussion

### 3.1. Analysis of Methylation Activity in C. carboxidivorans

Undiscovered RM systems can be predicted based on genome sequences by screening for homology to genes representing known restriction enzymes and MTases, which are collected in the online resource REBASE (http://rebase.neb.com; [31]). Using this approach, ten restriction enzyme genes were predicted in the *C. carboxidivorans* P7 genome, representing three Type I RM systems, two Type II systems, two Type III systems, and three Type IV systems (Table 1). The *C. carboxidivorans* genome encodes an unusually large number of restriction enzymes compared to other *Clostridium* species (Table 2). Furthermore, eight MTase genes correlating with the Type I, II, and III restriction enzymes, as well as one additional orphan Type II MTase were predicted for *C. carboxidivorans*. Two of the Type II restriction enzymes and MTases were present as fusion proteins. 

**Figure A1:**
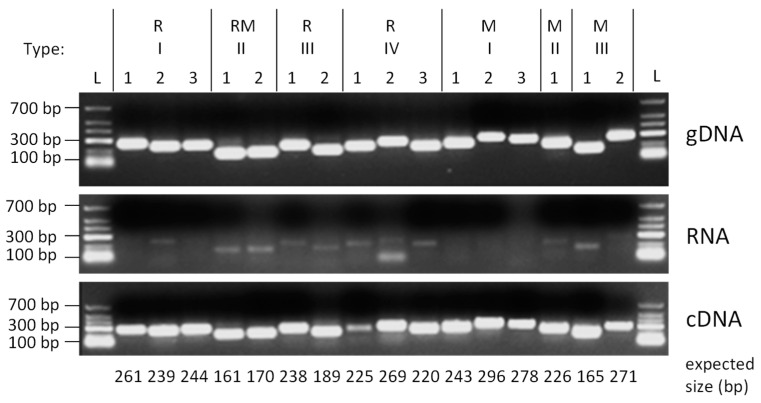
Verification of the transcription of predicted RM genes in *C. carboxidivorans*. The predicted RM genes are shown in Table 1. Total RNA was extracted from exponentially growing *C. carboxidivorans* cells. After two DNase treatments, gene-specific primers were used to amplify all RM-related genes in the *C. carboxidivorans* genome. Lanes: L—GeneRuler Low Range DNA Ladder, R—restriction enzyme (subunit); M—methyltransferase; RM—fused restriction enzyme and methyltransferase. Type indicates the designation of the RM system as Type I, II, III, or IV. Number refers to the short name used in Table 1. For example, the first sample lane on the left is a PCR product for the primers specific to Type I restriction enzyme 1.

To determine whether the predicted RM genes in *C. carboxidivorans* P7 were expressed, the total RNA was isolated, followed by two DNase treatments to destroy any genomic DNA contamination that could lead to false positive results. The RNA was then reverse transcribed into cDNA and amplified using primers specific to each predicted RM component (Table A3). We used genomic DNA as a positive control for the RM genes and RNA, following the DNase step before cDNA synthesis, as a control for DNA contamination. After 35 amplification cycles, the RNA negative control lane contained only weak bands, confirming that the samples contained negligible amounts of residual genomic DNA (Table A1). In contrast, all of the cDNA lanes produced strong bands, indicating that all of the RM components in the *C. carboxidivorans* genome were expressed under our selected experimental conditions (Figure A1). Prior to this study, methylation patterns in the *C. carboxidivorans* genome were partially predicted but not experimentally verified. To identify the comprehensive set of native *C. carboxidivorans* RM motifs, we isolated genomic DNA from heterotrophic cultures for PacBio sequencing and methylation analysis. 

During SMRT sequencing, labeled nucleotides are polymerized on the complementary DNA strand. Delays in nucleotide incorporation caused by base modifications such as methylation increase the gap between fluorescence pulses, known as the inter-pulse duration. Different modifications affect polymerase kinetics in specific ways, enabling a simultaneous readout of the primary nucleotide sequence and certain base modifications [29]. It is therefore possible to detect m^6^A (N^6^-methyladenosine) and m^4^C (N^4^-methylcytosine) even with a low sequencing coverage [33]. 

**Figure A2:**
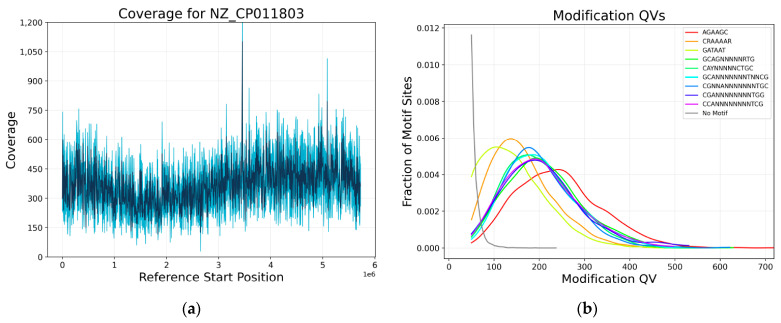
Coverage display and modification quality values (modQVs) from *C. carboxidivorans* P7 PacBio sequencing data. (**a**) PacBio sequencing depth obtained by read mapping onto the *C. carboxidivorans* reference genome NZ_CP011803 (**b**) Overview of the modification QVs of the different motif sites detected.

The coverage diagram for the circular *C. carboxidivorans* genome revealed high coverage between 4 and 5 Mbp of the reference sequence and low coverage between 1 and 2 Mbp (Figure A2a). This matches the Z-shaped curve obtained for actively growing cultures and the predicted position of the *oriC* region between positions 4,610,600 and 4,611,172 in the reference sequence [34]. All motifs detected by PacBio sequencing contained m^6^A residues, with no evidence of m^4^C cytosine methylation. We also screened for putative m^5^C (5-methylcytosine) sites using bisulfite treatment followed by sequencing on an Illumina NovaSeq instrument in PE150 mode.

**Figure 1:**
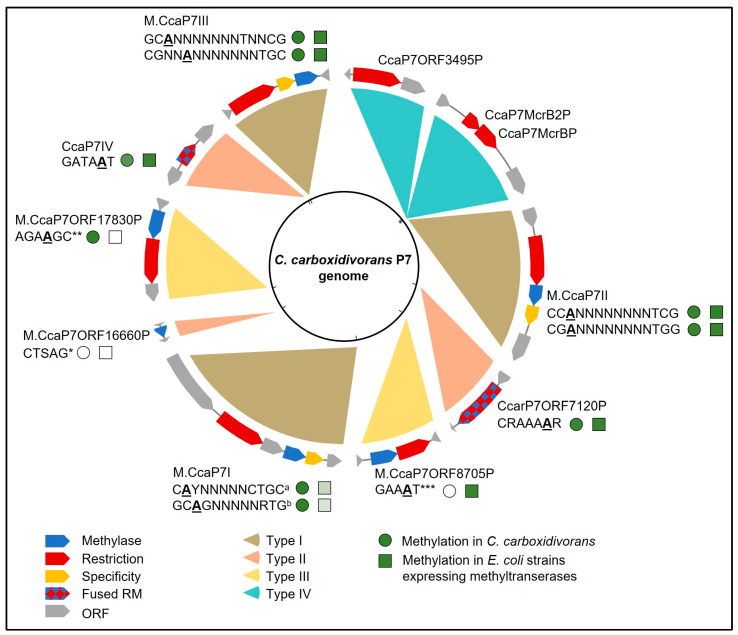
Schematic map of the *Clostridium carboxidivorans* P7 genome showing genes encoding RM system components and the results of methylome analysis in *C. carboxidivorans* and *E. coli* strains expressing the corresponding methyltransferases. Genes representing RM systems are color coded according to their assumed functional unit and flanking genes are shown in gray. The type of the RM system is shown as a colored segment (image adapted and modified from REBASE). Each methyltransferase gene is accompanied by an assigned recognition motif. Methylated bases are underlined and in bold (**A**). The percentage of methylated motifs in *C. carboxidivorans* and *E. coli* strains expressing the corresponding methyltransferases is visualized by the intensity of the colored symbols (○ and □, respectively). Genomic DNA isolated from cells at the late exponential/early stationary growth phase was analyzed using PacBio sequencing and sequencing after bisulfite conversion. More details are provided in Table A4. * The predicted motif CTSAG for MTaseII.1 (Table 1) was not detected in *C. carboxidivorans* or in the *E. coli* expression strains we investigated, but the gene was transcribed in *C. carboxidivorans* (Figure A1). ** The motif AGA**A**GC was detected in *C. carboxidivorans* but not in the *E. coli* expression strains. Because all other MTases were accounted for, this motif is probably recognized by Mtase III.1. *** The motif GAA**A**T was detected in *E. coli* but not in *C. carboxidivorans*. In the *E. coli* strain expressing the MTase and the specificity unit of M.CcaP7I, the precise motifs were not found but very similar motifs (^a^ tC**A**YbNNNNCTGC and ^b^ GC**A**GNNNNNRTGnnnh, with differing bases in lower case) were detected using a lower modification threshold (Qmod) score of 25.

Bisulfite treatment converts unmethylated cytosine to thymidine, whereas methylated cytosine and other bases remain unaffected, allowing the position of m^5^C sites to be confirmed by comparing the bisulfite sequence to the reference sequence. We found no evidence of m^5^C cytosine methylation, suggesting that all RM systems in *C. carboxidivorans* introduce m^6^A modifications. These data have been deposited in the NCBI sequence read archive (BioProject-No. PRJNA994489). The results are summarized in Figure 1 and are set out in more detail in Table A4. The PacBio analysis of *C. carboxidivorans* genomic DNA revealed nine methylation motifs (Figure A2b). Three of them were pairs of non-palindromic complementary sequences, which are methylated by one enzyme each (grouped in Table A4). 

However, we did not find the predicted motif CTSAG for the Type II.1 MTase. Given that the corresponding native gene was expressed (Figure A1), we assume that the quantity of the transcript was insufficient to produce enough enzyme, or that the enzyme was inactive. Furthermore, the sequences GATA**A**T, CRAAA**A**R, and AGA**A**GC were methylated at high frequencies of 84.8%, 97.3%, and 99.6%, respectively. Methylome analysis in *C. carboxidivorans* therefore confirmed the activity of six MTases, but it was still necessary to determine which of the eight predicted MTases recognize which of the six identified motifs and which enzymes are inactive.

### 3.2. Expression of C. carboxidivorans MTases for In Vivo Methylation in E. coli

**Figure A3:**
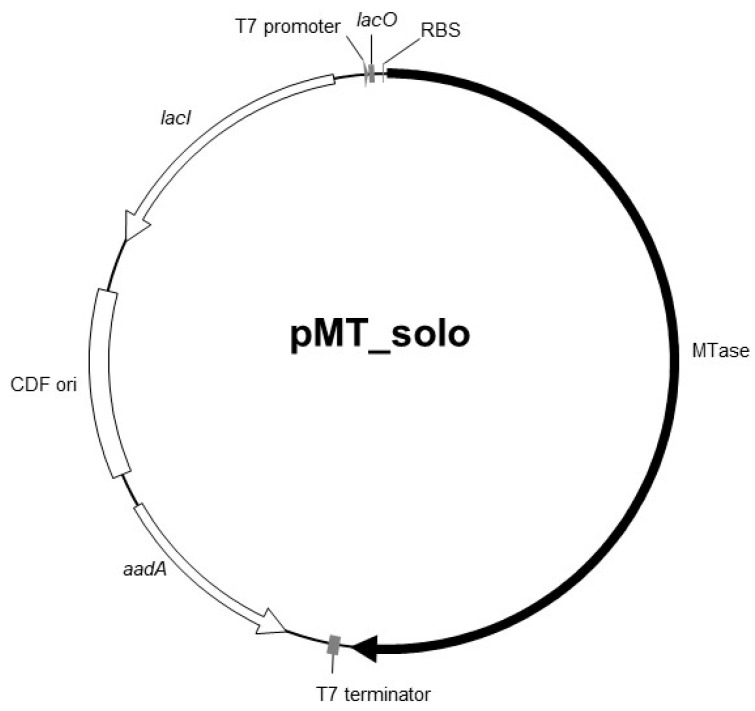
Representative plasmid map of MTase expression vectors for the investigation of *C. carboxidivorans* MTase recognition motifs. The plasmid is based on pCDFDuet-1, with a lactose/IPTG-inducible T7 promoter. The second open reading frame, including its promoter and ribosome binding site (RBS), was removed from this construct. The black arrow indicates the location of the MTase gene. Abbreviations: *lacI*—lactose inhibitor, controlling the activity of the T7 promoter (by binding the *lacO* sequence); *lacO*—lac operator; CDF ori—CloDF13 origin of replication; *aadA* (*smR*)—streptomycin/spectinomycin resistance gene.

In order to pair the eight *C. carboxidivorans* MTases with their specific target sites, we inserted each methyltransferase gene into the expression vector pCDFDuet-1 under the control of the lactose-inducible T7 promoter for the transformation of *E. coli* NEB 5-alpha cells. To ensure sequence recognition by the Type I MTases, these genes were co-expressed with the corresponding specificity subunit but not the restriction subunit. A schematic representation of one of the resulting pMT_solo plasmids, named according to the specific MTase, is shown in Figure A3. Following the overnight induction of expression with 2 mM IPTG, total DNA was isolated from all eight *E. coli* strains for PacBio sequencing to annotate the putative recognition motifs associated with each MTase (Figure 1 and Table A4). Almost all of the detected motifs were methylated at a frequency of nearly 100%. The exception was Type I.1 M.CcarP7I, where we detected two complementary motifs only when using a lower modification threshold (tC**A**YbNNNNCTGC and GC**A**GNNNNNRTGnnnh). In *E. coli*, these motifs were modified at frequencies of 32.0% and 20.6%, respectively, indicating low enzyme activity (or low expression). In contrast, both motifs were modified at a frequency of nearly 100% by the native enzyme in *C. carboxidivorans* (Table A4). These results confirmed that most *C. carboxidivorans* MTases were active in the *E. coli* expression strains. Recognition motifs could be identified for all MTases encoded in the *C. carboxidivorans* genome by combining the predicted motifs from REBASE with the methylome data from *C. carboxidivorans* and the *E. coli* strains expressing single MTases (Table A4).

REBASE allows a comprehensive search of organisms with similar motifs when a particular recognition sequence is used as a search query. The motifs detected by methylome analysis were therefore used to search REBASE for matches in other organisms (Table 3).

The complementary pair C**A**YNNNNNCTGC/GC**A**GNNNNNRTG did not match any other organisms, whereas the pair CC**A**NNNNNNNNTCG/CG**A**NNNNNNNNTGG is also found in *Vibrio harveyi* NCTC12970 (as part of the slightly longer motif CC**A**NNNNNNNNTCGT/ACG**A**NNNNNNNNTGG) and the pair GC**A**NNNNNNNTNNCG/CGNN**A**NNNNNNNTGC is also found in *Klebsiella pneumoniae* AR_0139 (as part of the longer motif GGC**A**NNNNNNNTNNCG/CGNN**A**NNNNNNNTGCC). The Type II MTase motif GATA**A**T matched several strains of *C. botulinum* and *C. sporogenes*. Although we found no matches for the motif CRAAA**A**R in REBASE, a recently published article discussing the complete genome sequence of *Clostridium cadaveris* IFB3C5 reported the same motif [35]. However, the non-generalized (more specific) recognition sequence CAAAA**A**R was detected multiple times in the genus *Clostridium*, including in *C. botulinum* and *C. sporogenes*; in others such as *C. pasteurianum*, *C. tetani*, *C. autoethanogenum* [38,39], and *C. ljungdahlii* [36,39]; and even in *Eubacterium limosum* B2 [37] and *Acetobacterium woodii* DSM1030 [39]. A closely related motif (CAAAA**A**) was found in *Clostridium difficile* 630. In a study of 36 different *C. difficile* strains, the motif CAAAA**A** was ubiquitously methylated and was proposed to have a conserved function influencing sporulation [25]. Due to their high similarity, the motifs CRAAA**A**R and CAAAA**A**R might have the same function.

The predicted Type II MTase target site CTSAG was not modified in *C. carboxidivorans* or in the *E. coli* strain expressing the corresponding enzyme. However, when we screened REBASE with this sequence, we recovered 263 entries representing many different species, including *C. botulinum* and *C. perfringens*. Furthermore, *K. pneumoniae* NCTC9151 carries a Type III restriction enzyme with this recognition sequence, and the motif is also found in *Lactobacillus acidophilus* and *Bacillus stearothermophilus* Isl 15-111. In the latter case, the corresponding MTase gene was cloned and its enzyme activity was verified [40]. Those characterized enzymes are classified in REBASE as gold standard enzymes. Given that no corresponding restriction enzyme was predicted for this MTase in *C. carboxidivorans*, it may have evolved to fulfil a different, perhaps regulatory function. Alternatively, it might be active only under specific growth conditions that were not tested in this study. Finally, it may have lost its function after the corresponding restriction enzyme was lost from the genome, thus removing the selection pressure for CTS**A**G methylation.

The Type III MTase motif AGA**A**GC was the only motif that was methylated in *C. carboxidivorans* but not in *E. coli*. The only other organism to match this motif in REBASE was *Lactococcus lactis* ssp. *lactis* strain UC073. Interestingly, the second Type III MTase motif GAA**A**T was only methylated in *E. coli* but not in *C. carboxidivorans*. PacBio data confirmed that the motif is also methylated in *Arachnia propionica* F0231, *Corynebacterium diphtheriae* NCTC10838, *Helicobacter fennelliae* NCTC13102, and *Pseudopropionibacterium propionicum* NCTC11666, but the corresponding enzymes have not yet been assigned. 

The diverse results arising from the comparison of methylation in different bacteria raises some interesting questions. Whereas some motifs, such as the one recognized by Type II.1, were shared by a very heterogeneous group of organisms, others, such as the one recognized by Type II RM.1, were limited to selected *Clostridium* species and the Type I.1 motif was not listed in the database at all. Species in the same genus as *C. carboxidivorans* were present, but also Gram-negative species such as *K. pneumoniae* and *V. harveyi*. This phylogenetically and environmentally diverse group sharing methylation motifs with *C. carboxidivorans* suggests that MTase genes may have been acquired by horizontal gene transfer or convergent evolution. Further studies are required to determine which of these scenarios is most likely.

## 4. Conclusions

The *C. carboxidivorans* genome encodes an unusually large number of active RM enzymes that are likely to hinder the genetic modification of this species. By defining the restriction motifs and corresponding MTases, it should be possible to develop reliable transformation protocols for *C. carboxidivorans*, as already shown for other *Clostridium* species. These methods include restriction alleviation, in which plasmid sequences are designed to avoid recognition motifs or plasmids are specifically methylated [12,26,41,42]. The analysis of RM systems in *C. carboxidivorans* provides important knowledge that will allow this species to be tailored for the industrial utilization of syngas. We observed interesting similarities between recognition motifs in *C. carboxidivorans* and distantly related bacteria from different habitats. Future research should consider how these RM traits were acquired, including convergent evolution and horizontal gene transfer, to provide insight into the evolution of RM systems in diverse ecosystems.

## Figures and Tables

**Table 1:** Restriction modification (RM) systems predicted in the genome of *C. carboxidivorans* P7. The table shows the RM type, gene names representing individual subunits, predicted recognition motifs (if available), genome coordinates, and the short names used for individual enzymes in this study. The genome sequence was obtained from GenBank (CP011803, 5,732,880 bp). Table modified from http://tools.neb.com/genomes/report.php?genome_id=20798 (accessed on 2 September 2021) based on the published *C. carboxidivorans* genome sequence [32].

Type	Subunit	Gene Name	Predicted Recognition Site	Coordinates	ORF (bp)	Short Name
I	S	S.CcaP7I	CAYNNNNNCTGC	2727394–2728617 c	1224	Type I.1
	M	M.CcaP7I	CAYNNNNNCTGC	2728619–2730115 c	1497	
	R	CcaP7IP	CAYNNNNNCTGC	2731721–2735029 c	3309	
I	R	CcaP7IIP	CCANNNNNNNNTCG	827150–830422	3273	Type I.2
	M	M.CcaP7II	CCANNNNNNNNTCG	830425–831846	1422	
	S	S.CcaP7II	CCANNNNNNNNTCG	831846–833189	1344	
I	R	CcaP7IIIP	GCANNNNNNNTNNCG	5411396–5414605	3210	Type I.3
	S	S.CcaP7III	GCANNNNNNNTNNCG	5414864–5416072	1209	
	M	M.CcaP7III	GCANNNNNNNTNNCG	5416106–5417695	1590	
II	M	M.CcaP7ORF16660P	CTSAG	3757440–3758204 c	765	Type II.1
II	RM	CcaP7IV	GATAAT	5299217–5300845	1629	Type II RM.1
II	RM	CcaP7ORF7120P	-	1647209–1650934	3726	Type II RM.2
III	R	CcaP7ORF17830P	-	4003447–4006389 c	2943	Type III.1
	M	M.CcaP7ORF17830P		4006403–4008355 c	1953	
III	R	CcaP7ORF8705P	-	2023100–2025358 c	2259	Type III.2
	M	M.CcaP7ORF8705P		2025352–2027229 c	1878	
IV	R	CcaP7ORF3495P	-	805395–808574	3180	Type IV.1
IV	R	CcaP7McrB2P	-	815415–816887	1473	Type IV.2
IV	R	CcaP7McrBP	-	816865–818568	1704	Type IV.3

c—complement.

**Table 2:** Comparison of genome sizes and the number of putative restriction enzymes (REs) and methyltransferase (MTases) encoded in the genomes of different *Clostridium* species. Data were retrieved from REBASE (http://rebase.neb.com, accessed 24 February 2023).

Organism	Genome Size (bp)	Accession Number	PacBio	Putative REs	Putative MTases
*Clostridium acetobutylicum* ATCC 824	3,940,880	AE001437 (NC_003030)	No	3	6
*Clostridium autoethanogenum* DSM 10061	4,352,446	CP012395	Yes	6	4
*Clostridium beijerinckii* NCIMB 8052	6,000,632	CP000721 (NC_009617)	No	2	2
*Clostridium botulinum* A ATCC 19397	3,863,450	CP000726 (NC_009697)	Yes	2	5
*Clostridium carboxidivorans* P7	5,732,880	CP011803	Yes	10	8
*Clostridium cellulolyticum* H10	4,068,724	CP001348 (NC_011898)	No	4	10
*Clostridium cellulovorans* 743B	5,262,222	CP002160 (NC_014393)	No	6	13
*Clostridium difficile* 630	4,290,252	AM180355 (NC_009089)	Yes	2	5
*Clostridium diolis* DSM 15410	5,940,808	CP043998	Yes	2	1
*Clostridium kluyveri* DSM 555	3,964,618	CP000673 (NC_009706)	No	5	13 (2 ^a^)
*Clostridium ljungdahlii* DSM 13528	4,630,065	CP001666 (NC_014328)	Yes	5	7
*Clostridium pasteurianum* BC1	4,990,707	CP003261	No	4	5
*Clostridium pasteurianum* DSM 525 = ATCC 6013	4,352,852	CP013018	Yes	4	8
*Clostridium perfringens* ATCC 13124	3,256,683	CP000246 (NC_008261)	Yes	6	7
*Clostridium sporogenes* DSM 795	4,142,990	CP011663	Yes	2	4
*Clostridium thermocellum* ATCC 27405	3,843,301	CP000568 (NC_009012)	Yes	5	11

^a^ The number in brackets shows the additional number of predicted putative MTases located on *C. kluyveri* DSM 555 plasmid pCKL555A [CP000674]. No additional MTases or restriction enzymes were predicted on *C. carboxidivorans* plasmid p19 [CP011804].

**Table 3:** MTase recognition motifs in *C. carboxidivorans* and their conservation in other bacterial species. Data were retrieved from REBASE (accessed 24 February 2023).

Methylation Motif	Type	Name	Homology
CAYNNNNNCTGC	Type I.1	M.CcaP7I	None
GCAGNNNNNRTG			
CCANNNNNNNNTCG	Type I.2	M.CcaP7II	Motif included in CCANNNNNNNNTCGT/ ACGANNNNNNNNTGG found in *Vibrio harveyi* NCTC12970
CGANNNNNNNNTGG			
GCANNNNNNNTNNCG	Type I.3	M.CcaP7III	Motif included in GGCANNNNNNNTNNCG/ CGNNANNNNNNNTGC found in *Klebsiella pneumoniae* AR_0139
CGNNANNNNNNNTGC			
CTSAG *	Type II.1	M.CcaP7ORF16660P	Predicted for many strains (including Clostridia) and several confirmed by PacBio, but also other species such as *Klebsiella pneumoniae* NCTC9151, *Lactobacillus acidophilus*, and *Bacillus stearothermophilus* Isl 15-111 (with gold standard enzyme)
GATAAT	Type II RM.1	CcaP7IV	Several strains from *Clostridium botulinum* and *Clostridium sporogenes*
CRAAAAR	Type II RM.2	CcaP7ORF7120P	Motif CRAAAAR: *Clostridium cadaveris* IFB3C5 ^a^ Motif CAAAAAR: several strains from *Clostridium botulinum* and *Clostridium sporogenes*; other species such as *Clostridium pasteurianum*, *Clostridium tetani*, *Clostridium autoethanogenum, Clostridium ljungdahlii* ^b^, *Eubacterium limosum* B2 ^c^ and *Acetobacterium woodii* DSM 1030 Motif CAAAAA: *Clostridium difficile* 630 (with gold standard enzyme)
AGAAGC **	Type III.1	M.CcaP7ORF17830P	*Lactococcus lactis* subsp. lactis strain UC073
GAAAT ***	Type III.2	M.CcaP7ORF8705P	*Pseudopropionibacterium propionicum* NCTC11666 *Corynebacterium diphtheriae* NCTC10838 *Helicobacter fennelliae* NCTC13102 *Arachnia propionica* F0231

* The methylation of motif CTSAG for MTaseII.1 (Table 1) was not detected in *C. carboxidivorans* or in the *E. coli* expression strains in this study, but the native gene was expressed in *C. carboxidivorans* (Table A1) ** The motif AGA**A**GC was detected in *C. carboxidivorans* but not in the *E. coli* expression strains. However, because all the other MTases were accounted for, this motif is probably recognized by MTase III.1. *** The motif GAA**A**T was detected in *E. coli* but not in *C. carboxidivorans*. Other organisms with similar recognition motifs were found by screening REBASE. Further data taken from the literature: ^a^ [35], ^b^ [36], ^c^ [37].

## Data Availability

Data is contained within the article or in Appendix A. The *C. carboxidivorans* methylome data were deposited at NCBI SRA (BioProject-No. PRJNA994489) and will be made available after publication. Further data will be made available on reasonable request.

## Appendix A

**Table A1:** List of *Escherichia coli* strains used in this study.

Strain Name		Properties
*E. coli*	NEB 5-alpha ^a^	Strain for cloning of plasmids
*E. coli*	soloI.1	Strain with MTase I.1 (S.CcaP7I + M.CcaP7I)
*E. coli*	soloI.2	Strain with MTase I.2 (M.CcaP7II + S.CcaP7II)
*E. coli*	soloI.3	Strain with MTase I.3 (S.CcaP7III + M.CcaP7III)
*E. coli*	soloII.1	Strain with MTase II.1 (M.CcaP7ORF16660P)
*E. coli*	soloIIRM1	Strain with MTase and fused restriction enzyme IIRM1 (CcaP7IV)
*E. coli*	soloIIRM2	Strain with MTase and fused restriction enzyme IIRM2 (CcaP7ORF7120P)
*E. coli*	soloIII.1	Strain with MTase III.1 (M.CcaP7ORF17830P)
*E. coli*	soloIII.2	Strain with MTase III.1 (M.CcaP7ORF8705P)

^a^ Provided by New England Biolabs (Ipswich, MA, USA).

**Table A2:** List of primers used for the cloning of CMTase genes in pMT_solo constructs.

Solo MTs	Primer Sequence (5′–3′)	Anneals
pMT1-3 fw (pCDF1-3 fw)	TTAACCTAGGCTGCTGCCACCGCTGAGCAATAACTAGC	pCDFDuet backbone
pCDF Duet rv neu	CATGGTATATCTCCTTATTAAAGTTAAACAAAATTATTTCTAC	pCDFDuet backbone
TypeIII MT1_fwd	TAATAAGGAGATATACCATGGCTAACTTAATTGAAAAC	*C. carboxidivorans* Type III M1
TypeIII MT1_rev	GTGGCAGCAGCCTAGGTTAATATCCTATATACTCATAATATCTT TTATATCATTTC	*C. carboxidivorans* Type III M1
TypeIII MT2_fwd	TGTTTAACTTTAATAAGGAGATATACCATGGAAAAAGTATATG CATTTG	*C. carboxidivorans* Type III M2
TypeIII MT2_rev	TTGCTCAGCGGTGGCAGCAGCCTAGGTTAAATGCCTACCATG ACTTTAG	*C. carboxidivorans* Type III M2
TypeII MT1_fwd	TGTTTAACTTTAATAAGGAGATATACCATTAGAAATAAAGTAA TAAATAAAGAGTGC	*C. carboxidivorans* Type II M1
TypeII MT1_rev	TTGCTCAGCGGTGGCAGCAGCCTAGGTTAAGCTCTAAACAAA TTTAAGCTG	*C. carboxidivorans* Type II M1
TypeII RM1_fwd	TGTTTAACTTTAATAAGGAGATATACCATGTATAAAGACGTTA AATTAGAAAAAAG	*C. carboxidivorans* Type II R+M 1
TypeII RM1_rev	TTGCTCAGCGGTGGCAGCAGCCTAGGTTAATTCTTACAGCAGT TTATCTC	*C. carboxidivorans* Type II R+M 1
TypeII RM2_fwd	TGTTTAACTTTAATAAGGAGATATACCATGGATAAGACTAAAG TAAAATCC	*C. carboxidivorans* Type II R+M 2
TypeII RM2_rev	TTGCTCAGCGGTGGCAGCAGCCTAGGTTAAGTTAATTATATCT TAAAAAATAAATCCTTTTTAAC	*C. carboxidivorans* Type II R+M 2
TypeI.1_fwd	TGTTTAACTTTAATAAGGAGATATACCATGTTAAACAGCGAGA CAAAAAG	*C. carboxidivorans* Type I M+S 1
TypeI.1_rev	TTGCTCAGCGGTGGCAGCAGCCTAGGTTAATTAATTAAATAGT TCTCCTTTGAAAG	*C. carboxidivorans* Type I M+S 1
TypeI.2_fwd	TGTTTAACTTTAATAAGGAGATATACCATGAATACACAAGAGA TAGTAAG	*C. carboxidivorans* Type I M+S 2
TypeI.2_rev	TTGCTCAGCGGTGGCAGCAGCCTAGGTTAACTATATATCTTTAC TCAATATTTCCC	*C. carboxidivorans* Type I M+S 2
TypeI.3_fwd	TGTTTAACTTTAATAAGGAGATATACCATGGAAAAAAACAAA AATAAACC	*C. carboxidivorans* Type I M+S 3
TypeI.3_rev	TTGCTCAGCGGTGGCAGCAGCCTAGGTTAACTAAATTTTTACA CCTAAAATCTTCAATTG	*C. carboxidivorans* Type I M+S 3

**Table A3:** List of RT-PCR primers used in this study.

Primer	Primer Sequence (5′–3′)	Product Size (bp)	Anneals
RI.1_fw	AGTGAGCCTAGACAGGTTTG	261	*C. carboxidivorans* Type I R 1
RI.1_rv	CCAGCTTGCCTCCATTAATC		*C. carboxidivorans* Type I R 1
RI.2_fw	AGCAACAGTACAGGCTATGG	239	*C. carboxidivorans* Type I R 2
RI.2_rv	GGCTGGTGTAGCTGTAAGTG		*C. carboxidivorans* Type I R 2
RI.3_fw	AAGTGGCAGTTACGTTTAGC	244	*C. carboxidivorans* Type I R 3
RI.3_rv	AGTTCTGGTGCATCAAATCC		*C. carboxidivorans* Type I R 3
RMII.1_fw	AGAAAGAATAAGCAGTGCAAAG	161	*C. carboxidivorans* Type II R+M 1
RMII.1_rv	AGTCTATCAGGCAGTACAAATC		*C. carboxidivorans* Type II R+M 1
RMII.2_fw	GACATAGGAGCAAGGTATTGTC	170	*C. carboxidivorans* Type II R+M 2
RMII.2_rv	TCGCCTACCTGGATATTGTAAG		*C. carboxidivorans* Type II R+M 2
RIII.1_fw	TCGGCCTTAAGAGAAGGTTG	238	*C. carboxidivorans* Type III R 1
RIII.1_rv	TTACGCCACCATCTTCTTCG		*C. carboxidivorans* Type III R 1
RIII.2_fw	TGGATGGGATTGTCCGAGAG	189	*C. carboxidivorans* Type III R 2
RIII.2_rv	GAAAGGCTGCCGACTTTAAC		*C. carboxidivorans* Type III R 2
RIV.1_fw	AAGTGCTGGATAGAGCAAATAC	225	*C. carboxidivorans* Type IV R 1
RIV.1_rv	CAAACTGCATGTCACATTGTTC		*C. carboxidivorans* Type IV R 1
RIV.2_fw	TACACAGCTATTCGCAATGATG	269	*C. carboxidivorans* Type IV R 2
RIV.2_rv	TCCAGCCACTTTATTGTTTCAC		*C. carboxidivorans* Type IV R 2
RIV.3_fw	ATGGACTAGAGGCGGATATG	220	*C. carboxidivorans* Type IV R 3
RIV.3_rv	GCTGCTTTCTCCAAGTACTG		*C. carboxidivorans* Type IV R 3
MI.1_fw	AACCCATGTGCTGAGGATAAG	243	*C. carboxidivorans* Type I M1
MI.1_rv	GTTCATGGAGGCTATTCTAACC		*C. carboxidivorans* Type I M1
MI.2_fw	TGGCTCTTATGAATGCTATGC	296	*C. carboxidivorans* Type I M2
MI.2_rv	TATCTTTGTTCCGTCACCTTCC		*C. carboxidivorans* Type I M2
MI.3_fw	AGTAGTTACGAGCGGTGTAG	278	*C. carboxidivorans* Type I M3
MI.3_rv	GTGGATGGTCAATCCCTTTC		*C. carboxidivorans* Type I M3
MII.1_fw	TGCCTATGAAAGCACATGAAG	226	*C. carboxidivorans* Type II M1
MII.1_rv	TGTGTTTGGTGCAGAGGATAAG		*C. carboxidivorans* Type II M1
MIII.1_fw	GCTGCAGGGTATGAAAGTTG	165	*C. carboxidivorans* Type III M1
MIII.1_rv	AAGAGGCGATGGTCGTTTAG		*C. carboxidivorans* Type III M1
MIII.2_fw	GCAGCAGTACCAATTCTCAATC	271	*C. carboxidivorans* Type III M2
MIII.2_rv	AAAGTAACTCCTCCCTCAGAAG		*C. carboxidivorans* Type III M2

**Table A4:** Methylome analysis of *C. carboxidivorans* P7 and *E. coli* strains expressing corresponding MTases. Methylated bases are underlined and in bold (**A**). Gray rows indicate complementary motifs, where the same enzyme acts on complementary DNA strands. Genomic DNA was isolated from cells at the late exponential to early stationary phase and was analyzed using PacBio sequencing and bisulfite sequencing.

Methylation Motif	Type	Name	*C. carboxidivorans*					*E. coli*				
			% of Motifs Detected	# of Motifs Detected	# of Motifs in Genome	Mean QV	Mean Coverage	% of Motifs Detected	# of Motifs Detected	# of Motifs in Genome	Mean QV	Mean Coverage
CAYNNNNNCTGC ^a^	I.1	M.CcaP7I	99.5	1047	1052	201.2	177.0	32.0 ^a^	91 ^a^	284 ^a^	62.1 ^a^	209.6 ^a^
GCAGNNNNNRTG ^b^			99.5	1047	1052	212.9	177.0	20.6 ^b^	219 ^b^	1060 ^b^	59.4 ^b^	210.1 ^b^
CCANNNNNNNNTCG	I.2	M.CcaP7II	99.1	241	243	207.1	182.1	99.6	3126	3137	188.0	142.4
CGANNNNNNNNTGG			98.7	240	243	211.3	182.9	98.7	3097	3137	177.6	143.1
GCANNNNNNNTNNCG	I.3	M.CcaP7III	99.5	451	453	208.0	183.4	99.3	4075	4100	146.2	108.3
CGNNANNNNNNNTGC			99.5	451	453	201.3	183.3	99.4	4078	4100	144.9	108.0
CTSAG *	II.1	M.CcaP7ORF 16660P	-	-	-	-	-	-	-	-	-	-
GATAAT	II RM.1	CcaP7IV	84.8	6251	7367	152.9	179.7	98.2	3609	3672	248.7	211.1
CRAAAAR	II RM.2	CcaP7ORF 7120P	97.3	6321	6490	166.7	177.8	99.3	4368	4395	247.5	257.0
AGAAGC **	III.1	M.CcaP7ORF 17830P	99.6	4629	4647	245.0	180.4	-	-	-	-	-
GAAAT ***	III.2	M.CcaP7ORF 8705P	-	-	-	-	-	99.2	12,981	13,076	167.1	133.6

%—percent. #—number. * The predicted motif CTSAG for MTaseII.1 (Table 1) was not detected in *C. carboxidivorans* or in the *E. coli* expression strains, but the corresponding native gene was expressed in *C. carboxidivorans* (Figure A1). ** The motif AGA**A**GC was detected in *C. carboxidivorans* but not in the *E. coli* expression strains. Because all other MTases were accounted for, this motif is probably recognized by MTase III.1. *** The motif GAA**A**T was detected in *E. coli* but not in *C. carboxidivorans*. In the *E. coli* strain expressing M.CcaP7I MTase and its corresponding specificity unit, the exact motifs could not be found, but very similar motifs (^a^ tC**A**YbNNNNCTGC and ^b^ GC**A**GNNNNNRTGnnnh, differing bases are in lower case) were detected at a lower modification threshold (Qmod) score of 25.
